# Supplementary material for: Bayesian refinement of protein structures and ensembles against SAXS data using molecular dynamics
Source: PLoS Comput Biol. 2017 Oct 18;13(10):e1005800. doi: 10.1371/journal.pcbi.1005800 (PMC5662244; doi:10.1371/journal.pcbi.1005800)
Supplement: S1 Archive — (BZ2) [file pcbi.1005800.s011.tar.bz2 › gromacs_4.62_bayesian_saxs_refi/share/html/online/g_helix.html]

g\_helix


| g\_helix Main Table of Contents |

**VERSION 4.6  
Sat 19 Jan 2013**

---

### Description

g\_helix computes all kinds of helix properties. First, the peptide
is checked to find the longest helical part, as determined by
hydrogen bonds and φ/ψ angles.
That bit is fitted
to an ideal helix around the z-axis and centered around the origin.
Then the following properties are computed:

**1.** Helix radius (file radius.xvg). This is merely the
RMS deviation in two dimensions for all Cα atoms.
it is calculated as sqrt((sum\_i (x^2(i)+y^2(i)))/N) where N is the number
of backbone atoms. For an ideal helix the radius is 0.23 nm  
**2.** Twist (file twist.xvg). The average helical angle per
residue is calculated. For an α-helix it is 100 degrees,
for 3-10 helices it will be smaller, and
for 5-helices it will be larger.  
**3.** Rise per residue (file rise.xvg). The helical rise per
residue is plotted as the difference in z-coordinate between Cα
atoms. For an ideal helix, this is 0.15 nm  
**4.** Total helix length (file len-ahx.xvg). The total length
of the
helix in nm. This is simply the average rise (see above) times the
number of helical residues (see below).  
**5.** Helix dipole, backbone only (file dip-ahx.xvg).  
**6.** RMS deviation from ideal helix, calculated for the Cα
atoms only (file rms-ahx.xvg).  
**7.** Average Cα - Cα dihedral angle (file phi-ahx.xvg).  
**8.** Average φ and ψ angles (file phipsi.xvg).  
**9.** Ellipticity at 222 nm according to Hirst and Brooks.

### Files

| option | filename | type | description |
| --- | --- | --- | --- |
| **-s** | topol.tpr | Input | Run input file: tpr tpb tpa |
| **-n** | index.ndx | Input | Index file |
| **-f** | traj.xtc | Input | Trajectory: xtc trr trj gro g96 pdb cpt |
| **-to** | gtraj.g87 | Output, Opt. | Gromos-87 ASCII trajectory format |
| **-cz** | zconf.gro | Output | Structure file: gro g96 pdb etc. |
| **-co** | waver.gro | Output | Structure file: gro g96 pdb etc. |

### Other options

| option | type | default | description |
| --- | --- | --- | --- |
| **-[no]h** | bool | no | Print help info and quit || **-[no]version** | bool | no | Print version info and quit || **-nice** | int | 19 | Set the nicelevel || **-b** | time | 0 | First frame (ps) to read from trajectory || **-e** | time | 0 | Last frame (ps) to read from trajectory || **-dt** | time | 0 | Only use frame when t MOD dt = first time (ps) || **-[no]w** | bool | no | View output .xvg, .xpm, .eps and .pdb files || **-r0** | int | 1 | The first residue number in the sequence || **-[no]q** | bool | no | Check at every step which part of the sequence is helical || **-[no]F** | bool | yes | Toggle fit to a perfect helix || **-[no]db** | bool | no | Print debug info || **-prop** | enum | RAD | Select property to weight eigenvectors with. WARNING experimental stuff: RAD, TWIST, RISE, LEN, NHX, DIP, RMS, CPHI, RMSA, PHI, PSI, HB3, HB4, HB5 or CD222 || **-[no]ev** | bool | no | Write a new 'trajectory' file for ED || **-ahxstart** | int | 0 | First residue in helix || **-ahxend** | int | 0 | Last residue in helix |

---

http://www.gromacs.org  
gromacs@gromacs.org
